# Investigating the impact of Cognitive Processing Therapy (CPT) on Post-Traumatic Stress Disorder (PTSD), depression, and anxiety symptoms in female victims of domestic violence

**Published:** 2024-07

**Authors:** Malihe Shirzadfard Jahromi, Masoume Ghazanfarpour, Aliakbar Haghdoost, Atefeh Ahmadi

**Affiliations:** ^*a*^ Student Research Committee, Razi Faculty of Nursing and Midwifery, Kerman University of Medical Sciences, Kerman, Iran.; ^*b*^ Nursing Research Center, Kerman University of Medical Sciences, Kerman, Iran.; ^*c*^ Modeling in Health Research Center, Institute for Futures Studies in Health, Kerman University of Medical Sciences, Kerman, Iran.; ^*d*^ Reproductive and Family Health Research Center, Kerman University of Medical Sciences, Kerman, Iran.

**Keywords:** Domestic Violence, Anxiety, Depression, Post-Traumatic, Stress Disorder, Cognitive, Processing Therapy

## Abstract

**Background:**

This study aimed to investigate the effects of CPT on PTSD, depression, and anxiety symptoms in female victims of domestic violence.

**Methods:**

A two-arm randomized clinical trial was conducted in Bandar-Abbas involving 62 female victims of domestic violence referred from private gynecology and obstetrics clinics. Initial screening for domestic violence was based on the World Health Organization violence questionnaire. Participants were randomly assigned to either a control group (n=32) or an intervention group (n=28) for a study duration of six months in 2022. Twelve group CPT sessions were conducted. The domestic violence questionnaire, Beck’s Depression Inventory, Beck’s Anxiety Inventory, and the Impact of Event Scale-Revised were completed in three time points: pre-test, post-test, and follow-up.

**Results:**

There was a statistically significant difference in the mean scores of depression, PTSD, and domestic violence (P less than .001) between the two groups; however, no significant difference was found in anxiety scores (P greater than .050).

**Conclusions:**

CPT is recommended for female victims of domestic violence to reduce symptoms of depression, PTSD, and domestic violence.

## Introduction

Violence against women is a severe form of gender discrimination which is any gender-based violence that results in sexual, painful, physical, or psychological harm to women. It may lead to forced deprivation of social or individual liberty.^1^ The most common type is domestic violence that one perpetrated by her intimate partner^2^ including sexual, physical, and emotional abuse and controlling behavior by the intimate partner. Domestic violence may lead to mental illnesses including depression, anxiety, PTSD^1^, distress, fear, sexual dysfunction, OCD^2^, suicidal thoughts, and SUD^3^.^3,4^

35% of women have experienced sexual and/or physical violence by their intimate partners or others.^5^ The prevalence of sexual and/or physical violence against women ranges from 14-17% among Brazilian women and up to 58.51% among Bolivian women.^6^ In Tanzania and Africa, the prevalence of sexual and/or physical intimate partner violence against women in their lifetime was 61%.^7^ Over fifty percent of married women in southwest Iran have reported experiencing domestic violence, with the mental form of abuse being the most prevalent.^8^ The annual prevalence of psychological, physical, and sexual violence was 60.9%, 34.7% and 37.7%, respectively. Self-employed, divorced and widowed, and less educated women were more likely to experience it.^9^ In the recent pandemic COVID-19, it increased significantly.^10^ The prevalence of severe TSD, depression and anxiety among women under domestic violence is about 41%, 33%, and 77%.^11,12^

Unwanted/unwise marriage, illiteracy/primary education, lower age, and previous marriage(s) were the significant risk factors for domestic violence.^13^ Physical violence is often accompanied by emotional and sexual violence.^14^ Since mental disorders due to domestic violence led to great suffering for victims, psychological interventions and treatments have been widely supported. 

In addition to medication, trauma-focused therapies such as Eye Movement, CPT, Prolonged Exposure Ther-apy, Desensitization, EMDR, and others with significant trauma focus are the current gold standard for the treatment of PTSD,^15^ but psychotherapy tends to provide greater and more long-lasting outcome improvements.^16^ A study aimed to compare the effectiveness of psycho-therapies for posttraumatic stress disorder in clinical practice showed CPT and PE^4^produce the same improvement.^17^ The research literature indicates that CPT is effective without preparatory treatment across a range of outcomes, settings, and populations, including clients with childhood trauma and comorbid conditions.^18^

CPT^5^is one of the cognitive therapies that has significantly improved PTSD and associated symptoms compared with other therapies. It was first proposed by Resick and Nishith (1992) to treat PTSD and depression associated with sexual abuse and consists of cognitive therapy and exposure by writing and reading about the traumatic event.^19^ The cognitive processing approach considers traumatic events as an experience that is inconsistent with one's beliefs about their selves, others, and the world.^20^ This treatment first focuses on thoughts such as suicide and denial and then on general beliefs about oneself and the world. The exposure part involves impelling patients to write the most traumatic part of the event and read it to themselves and the therapist; this encour-ages patients to understand their feelings deeply while reading and writing. Restructuring dysfunctional beliefs leads to a more balanced self-report.^21^

Intimate partner violence (IPV) creates psychological anguish, and victims need support, including psychotherapy, Cognitive Processing Therapy for the Treatment of PTSD, Depression, Anxiety Symptoms and Difficulties in Emotion Regulation in Syrian Refugee Women Exposed to Intimate Partner Violence showed CPT was a comprehensive therapeutic approach.^22^ CPT is an effective psychotherapy option for anxiety, PTSD, and MDD^6^.^23,24^ There are limited studies that investigate the efficacy of CPT on domestic violence, and there is no research in Iran with its specific culture that affects the mechanism and probability of CPT effectiveness.

Considering the positive features of CPT, including consistency with different cultures, flexibility, adaptability, and high efficiency for the treatment of domestic violence victims, the possibility of holding individual and group sessions, short duration of treatment, besides inadequacy of information on its effectiveness on domestic violence, the present study investigated the effect of CPT on the symptoms of PTSD, depression and anxiety in female victims of domestic violence.

1- Post-Traumatic Stress Disorder

2- Obsessive Compulsive Disorder

3-Substance Use Disorder 

4- Prolonged Exposure

5- Cognitive Processing Therapy

6- Major Depressive Disorder

## Methods 

**Setting: **The study population consisted of women referring to private gynecology and obstetrics clinics in Bandar-Abbas city in Iran during the first six months of 2022. The city was divided into three parts (center, north, and south), and the most crowded clinic in each of these areas, and a total of 62 women were selected using convenience sampling. All of the women were living with their husbands. Because the declaration of being under domestic violence a taboo in this culture, women under the affiliation of public health centers were not candidates to participate in the group therapy. Therefore, sampling was done from the private centers where people had more sense of privacy.

**Sample size calculation: **Minimum sample size was defined based on formula^25^ and less than 30 women were placed in the intervention group and 32 women in the control group. 

**Ethical consideration: **To conduct the study, after obtaining permission from the ethics committee (IR.KMU.REC.1397.509) of the esteemed Vice Chancellor for Research and Technology of Kerman University of Medical Sciences, the researcher was introduced to the clinic staff, the procedure was explained, necessary arrangements were made, and the staff support was attained. The control group could attend the same sessions after the intervention per their request. 

**Sampling: **It was an open label-study. After obtaining informed consent from the married women 288 completed violence assessment questionnaires. 134 questionnaires were positive for at least one of the subscales of domestic violence. All the women who met the inclusion criteria and were highly willing to participate in the intervention phase completed the related informed consent form and if they had any problem to fill the forms, they could ask the distributors who were trained about filling the instruments. Before the intervention, the BDI, BAI, and IES-R were completed to determine the mean scores of PTSD, depression, and anxiety. 

Moreover, determine the level of domestic violence, Mohseni Tabrizi domestic violence questionnaire (produced in Iran) was completed by the participants. Then considering the cut-off scores, individuals with severe depression, anxiety, or PTSD were referred to a psychologist and psychiatrist for further evaluation. Finally, 62 women were included in the study. The study population was randomly divided into two groups of control and intervention. Randomization was done through a random number table. 30 and 32 participants were placed into the intervention and control group, respectively. None of the control group members were aware of the content of the intervention and none of the intervention group members were aware of what was happening in another arm because there was no relationship and contact between two groups.

**Inclusion criteria: **Willingness to participate in the study, being over 18 years of age, not being addicted to narcotic or psychedelic drugs, and fluency in the Persian language.

** Exclusion criteria: **absence>2 sessions, experiencing psychological trauma and great stress, developing severe mental disorders requiring medication and immediate treatment. Individuals with scores > 35 in the Beck Depression Inventory (BDI), scores > 25 in the Beck Anxiety Inventory (BAI), and scores > 36 in the Impact of Event Scale–Revised (IES-R) in their pretest, and who were referred to a psychologist and psychiatrist (all of these scores are based on the instruments; cutoff points). 

**Figure 1:**
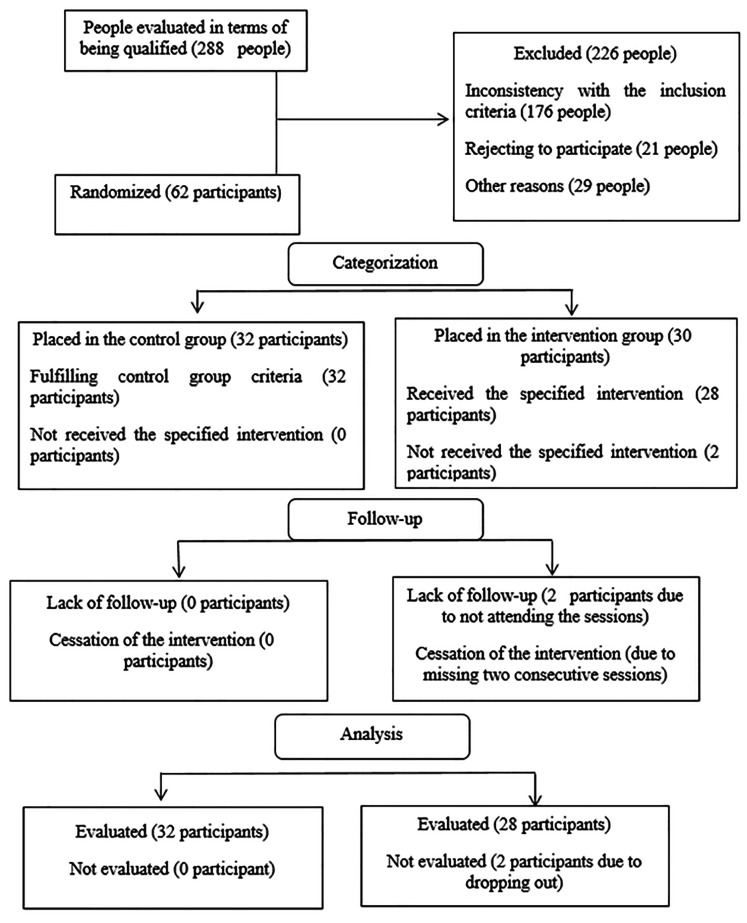
Consort flow chart of participant reporting

**Intervention procedure: **Treatment sessions for the intervention group were scheduled as group therapy in groups of 3 to 5 people. CPT was performed in twelve/90-minute sessions twice a week in the health center or the private center based on the convince of the group. No therapeutic intervention or counseling was done in the control group. Two participants in the intervention group were excluded from the study due to not attending two sessions. The participants in the control group did not meet any of the participants of the intervention group during the study (no data contamination) (Figure 1).

**CPT package: **Treatment sessions were designed based on the CPT therapeutic content for people with PTSD by Resick et al. (1992). The intervener was studying master of counseling in midwifery and had a certificate proving the capability of holding sessions. All of the intervention groups received the same intervention (Table 1 ).

**Table 1:** Summary of CPT Sessions of female victims of domestic violence.

Session number	Session content
Session 1	Introducing depression, PTSD, and anxiety symptoms and describing the treatment process
Session 2	Defining event, discussing the meaning of traumatic event that occurred to people, checking the homework, starting to identify the stuck point and related issues, introducing A-B-C worksheets explaining the relationship between thoughts and feelings
Session 3	Identifying thoughts and feelings, checking the homework, discussing stuck points by focusing on their similarities, investigating the event considering issues related to acceptance and self-blame, using Socratic questioning on stuck points
Session 4	Recalling the traumatic event and reading the full event report aloud by expressing emotions, identifying stuck points, helping the participants to challenge themselves with Socratic questioning, checking homework
Session 5	Reading the second report of the traumatic event, identifying the difference between the first and the second report, helping the participants to challenge themselves with Socratic questioning, helping the participants to challenge their stuck points, introducing the challenging questions worksheets
Session 6	Checking homework and the challenging questions worksheet, cognitive therapy related to stuck points, introducing the patterns of problematic thinking worksheet
Session 7	Checking homework, introducing the patterns of problematic thinking worksheet to further identify the stuck points associated with the trauma, introducing the safety issue, using Challenging Beliefs Worksheet to challenge safety beliefs
Session 8	Checking the challenging beliefs worksheet and helping the participants to challenge the problematic beliefs, Introducing the trust issue
Session 9	Checking homework and the challenging beliefs worksheet about the trust issue. Introducing the power/control issue.
Session 10	Checking homework and the challenging beliefs worksheet about the power/control issue. Introducing the esteem issue
Session 11	Reviewing homework and the challenging beliefs worksheet about the self-esteem unit, discussing the reaction to two assignments of giving and receiving compliments and engaging in a pleasing activity. Introducing the intimacy issue
Session 12	Checking homework and challenging beliefs worksheet about the intimacy issue, reading the last report of the traumatic event by the participants and the first one by the therapist and compare them, discussing stuck points of intimacy, identifying remained needs, encouraging continue the behavioral tasks related to giving and receiving compliments and doing good deeds for themselves, encouraging continue using the learnt skills

**Post-treatment assessment: **Immediately after the last treatment session and one month after it, the post-test questionnaires including BDI, BAI, IES-R, Mohseni Tabrizi domestic violence questionnaire were given to both groups and collected after completion.

**Tools**

***Demographic checklist: ***Demographic and contextual information questionnaire includes age, education, occupation, participant’s income, spouse’s income, number of children, children’s gender, years of marriage, age of the spouse, drug use, mental illness, husband's job, and spouse’s addiction.

***Domestic Violence Questionnaire (Mohseni Tabrizi): ***This is a 71-item tool related to patriarchal beliefs, spousal abuse, family upbringing, and learning about violence that investigates verbal, psychological, physical, and sexual violence. The scoring is based on the Likert scale (0-4) about violent behavior and the extent the person agrees with the violent behavior. 0-60, 60-120, and >120 indicate low, moderate, and high levels of domestic violence, respectively. Items and questions from previous studies conducted under the supervision of experienced people are used in Mohseni Tabrizi domestic violence questionnaire in Persian. The total Cronbach's alpha was 0.83.^26^

***Beck depression inventory (BDI^1^):*** This tool first created in 1961 by Beck, consists of 21 categories. These 21 items are arranged in the form of a series of statements, each of which is related to a specific symptom of depression. For each item, which is about one symptom of depression in this questionnaire, 4 to 6 statements are written indicating the mildest to the most severe symptoms. Quantitative values of each item are assigned from zero (indicating mental health in the corresponding item) to 3 (indicating severe disorder). Therefore, scores between 1 and 18, 19 and 28, 29 and 35, and 36 and 63 indicate no or minimal, mild, moderate, and severe depression, respectively. The internal consistency of BDI was 0.86 and the test-retest reliability of this questionnaire was reported to be 0.93 after a one-week interval (Beck et al., 1961). The results of the study in Persian context indicated that the internal consistency of the inventory was 0.87 and the test-retest reliability was 0.74.^27^

***Beck anxiety inventory (BAI^2^):*** BAI is a 21-item scale that specifically measures the severity of clinical anxiety symptoms in individuals and was proposed by Aaron T. Beck et al. Each item is about one of the most prevalent symptoms of anxiety (mental, physical, and fear symptoms) and scored 0-3. A total score of 0-7, 8-15, 16-25, and 26-63 indicate no or minimal, mild, moderate, and severe anxiety. The validity of the inventory was 0.92 and the reliability was reported to be 0.75 after a one-week interval.^28^ In the Persian context, the validity of 0.72, the reliability coefficient of 0.83 after a one-month interval, and an internal consistency of 0.92 were reported.^29^

***Impact of Event Scale - Revised (IES-R^3^) (Weiss & Marmar): ***This is a 22-item self-report scale that measures PTSD and includes the three main dimensions of intrusion, avoidance, and hyperarousal during the past 7 days. Items are scored from 0 to 4. A total score of 0-23, 24-32, 33-36, and above 47 is interpreted as no or minimal, mild, moderate, and severe PTSD, respectively.^30^ The validity of the questionnaire on the avoidance and intrusion scale was 0.85, and Cronbach's alpha was 0.87 and 0.84 on the avoidance and intrusion scale.^31^ For the Persian version of IES-R, Cronbach's alpha was 0.87 and 0.86 in the population between 10-20 and over 20 years old, and its test-retest method reliability was reported 0.86.^32^

**Data analysis**

In the present study, SPSS-22 software, the Chi-square tests, and the independent t-test were used to compare baseline variables in two groups. Furthermore, repeated measures ANOVA were used to compare the mean of PTSD, anxiety, depression, and violence variables obtained from different measurements in the two groups within the period of the study as a pretest, posttest (end of the last session), and follow up (one month later).^33^

1-Beck Depression Inventory

2-Beck Anxiety Inventory

3-Impact of Event Scale - Revised

## Results

**Characteristics of the sample: **The distribution of scores of all variables during pre-test, post-test, and 4-week follow-up for intervention and control groups was investigated. The homogeneity of absolute and relative frequency distributions of the studied groups was investigated using independent t-test along with Fisher’s and Chi-square tests. The distribution of demographic variables in the control and intervention groups is presented in Table 2. It can be seen that only women’s monthly income was significantly different between groups. The mean age of women was 29.46 (between 18 and 54 years), the mean years of marriage was 3.03 (between 1.5 and 15 years), the mean number of children was 1.32 (between 0 and 5 children), the mean age difference with spouse was 2.1 years younger than the husband (between 3 years older and 15 years younger). In addition, most of the women were housewives (80.10%). Only a small percentage of their spouses were unemployed (3.35%). 66.70% of the women had a diploma equivalent to 11 years of education (between 8 and 18 years) and 76.70% of their spouses had diploma or lower degrees (between 8 and 18 years). 18.33% of spouses were addicted to drugs (opium, methamphetamine or heroin). Women and their spouses’ monthly income (Dollar) is described in Table 3

**Table 2:** Demographic characteristics between intervention and control groups.

Demographic Characteristic	Intervention group	Control group	P-value
Age (year), mean (SD)	29.47 (7.39)	30.19 (6.36)	0.280*
Years of marriage, mean (SD)	3.04 (1.23)	3.03 (1.25)	0.520*
Number of children, mean (SD)	1.32 (1.32)	1.47 (1.46)	0.640*
Age difference with spouse, mean (SD)	2.11 (2.10)	2.47 (2.46)	0.310*
Woman monthly income (Dollar) mean (SD)	19.30 (1.07)	33.80 (1.51)	0.034
Spouse monthly income (Dollar) mean (SD)	156.25 (1.16)	161.45 (1.15)	0.657
Occupational status, n (%)	5 (17.91)	7(21.87)	0.760
Employment of spouse, n (%)	23 (82.14)	27(84.37)	0.420
Woman’s Diploma education and higher, n (%)	24 (85.70)	26(81.30)	0.724
Spouse’s diploma education and higher, n (%)	18 (64.30)	20(62.51)	0.124
Addicted spouse, n (%)	6 (21.43)	5(15.60)	0.898

Note: *:t-test, others: Chi-square

**Table 3:** Mean of changes and standard error of PTSD, depression, anxiety, and domestic violence scores in women during pre-test, post-test and follow-up in the control group.

Variable	Time		Intervention			Control			Time effect	Time* Group effect	Group effect
			Mean difference	SD	P-value	Mean difference	SD	P-value			
PTSD	Pre-test	Post-test	12.18	1.05	.001	0.97	0.84	.259	0.000	0.0001	*0.000 **0.014
	Pre-test	Follow	10.64	1.00	.001	0.72	0.62	.252			
	Post-test	Follow	-1.54	0.61	.018	-0.25	0.41	.550			
Depression	Pre-test	Post-test	7.61	0.78	.001	0.59	0.53	.274	0.000	0.0001	*0.000 ** 0.001
	Pre-test	Follow	5.79	0.66	.001	-0.81	0.36	.031			
	Post-test	Follow	-1.82	0.45	.001	-0.22	0.39	.577			
Anxiety	Pre-test	Post-test	2.39	0.94	.001	-1.91	0.55	.002	0.000	0.001	*0.000 **0.014
	Pre-test	Follow	1.75	0.41	.001	-0.78	0.38	.046			
	Post-test	Follow	-0.64	0.35	.077	1.13	0.51	.035			
Violence	Pre-test	Post-test	6.39	0.75	.001	0.78	0.38	.050	0.000	0.001	*0.031 **0.072
	Pre-test	Follow	4.36	0.80	.001	1.03	0.29	.001			
	Post-test	Follow	-2.04	0.45	.001	0.25	0.26	0.340			

*Posttest, **follow up

**Figure 2:**
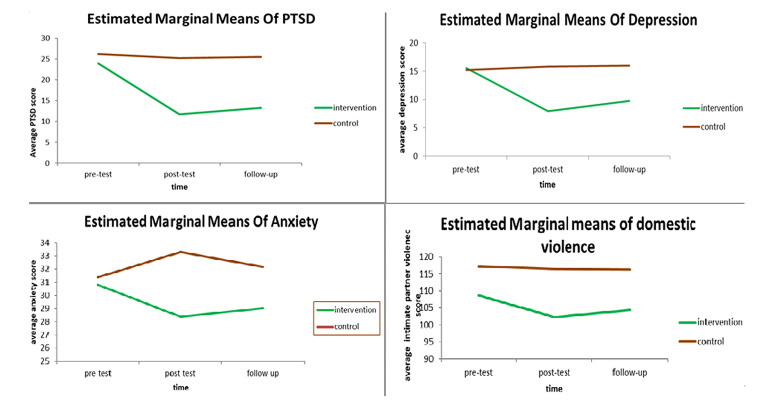
Comparison of the Mean Intimate Partner Violence, depression, anxiety and PTSD Scores in Intervention and Control Group During Three Months

**Intervention phase: **There is a significant difference in the changes in PTSD (P=0.014), depression (P=0.001) and anxiety (P=0.014) over time and between the intervention and control groups. The amount of domestic violence between the two groups showed a significant difference immediately after treatment (P=0.031) and a non-significant difference one month later (P=0.072) (Table 3 ,Figure 2).

## Discussion

In this study, for investigating the impact of CPT on the level of PTSD, depression and anxiety symptoms in female victims of domestic violence, group therapy was chosen because special emphasis is given to cohesion and the significant correlation between cohesion and outcome in group therapy.^34^ Cognitive Processing Therapy in a systematic review and meta-analysis showed its efficacy for PTSD, depression and anxiety.^33^ Interestingly, these methods is effective in serious mental health problems.^35^ Developmentally adapted CPT for PTSD after physical abuse showed improvement.^36^ The mechanism of changes in cognition via CPT is reducing feelings of guilt and self-blame as well as negative beliefs about the world and assimilated and overaccommodated stuck points to form a more balanced view of the trauma and also process natural emotions.^37^

In the present study, depression in female victims of domestic violence decreased significantly after the treatment in the post-test and follow up in the intervention group in line with some previous studies.^38^ Stress management techniques are helpful for control of depression.^39^ In CPT, the roots of our dysfunctional problems, cognitions, and thoughts are presented. Then, by performing planned techniques, the therapist tries to solve problems.^40^ The results of the present study in controlling anxiety are in line with the use of CPT in controlling depression, anxiety, phobia, OCD, paranoia in students exposed to trauma.^41^ This method also improved the symptoms of depression and anxiety in prisoners.^42^

Controlling PTSD in the recent research, is in line with one of the first studies on this method conducted by Resick and Nishith (1992) on the effectiveness of CPT on the PTSD associated with the effectiveness of this treatment in various groups of victims.^19^ Decreasing hopelessness as a positive mechanism in CPT, was shown to reduce the symptoms of PTSD. Feeling of hopelessness towards the future is a consequence of negative changes occur in thoughts and mood.^43^ CPT corrects cognitions about oneself, others, and the world towards the realistic cognitions, reduces other problematic thoughts, and improves symptoms of PTSD.^44^ Although reviewing the psychotraumatology showed different factors effect on the effectiveness of therapies on PTSD^45^ but group CPT increases social skills and sense of belongingness and autonomy^46^ when it is flexible, person centered with full care.^47^ Inconsistently, the various therapies examined do not completely rid patients of their issues with PTSD, but they have been found to significantly reduce the severity of the symptoms.^48^

CPT and prolonged exposure therapy were effective in treatment of PTSD; however, CPT was reported to be more effective in the betterment of some symptoms such as sense of guilt.^49,50^ One of the central points of this approach is the use of compassionate and create inner peace, security, and relief. This is also effective in treating those with feelings of shame and self-criticism as well as the ones who have difficulty feeling kind to themselves or others.^51^

In the present study, the rate of domestic violence significantly decreased by employing the techniques to decrease the probability of becoming a victim of violence and interpersonal harm.^52^ The relationship between domestic violence and mental disorder is undeniable in such a way that a decrease in the rate of domestic violence leads to a decrease in depression and PTSD. Conversely, a decrease in depression and PTSD levels reduces the possibility of becoming a victim of domestic violence.^53^ By re-experiencing past problems and painful situations^54^ as well as correcting dysfunctional thoughts, this method limits or removes cognitive distortions.^19,40,49^

Anxiety and depression of control group increased during this study. Furthermore, although there was improvement in posttest of variables in the intervention group, they increased in one-month. These results can be attributed to the presence of two unavoidable interfering factors during the study: the first factor could be the fact that the women were living with their husbands who were the perpetrators of domestic violence against them while most previous studies were conducted on women who were out of marital and traumatic relationships.^40,55^ Secondly, this study was run during the COVID-19 outbreak. According to a report of WHO (2020), emergency calls from people subjected to violence increased by 10-15% in some countries during the pandemic. In China, the reports of violence by an intimate partner have tripled. Women under traumatic relationships are more exposed to violence and dealing with stress. This level of violence during outbreaks can be associated with or exacerbate mental health disorders^56^ and domestic violence^57^ affected by staying in quarantine.^58,59^ Thirdly, the only significant different between two groups was women’s monthly income that was lower in intervention group. Women’s income was protective against IPV (intimate partner violence). ^59^ Therefore, the effect of CPT can be limited when is influenced by this factor. At last, although, sample size calculation was based on the sample size calculation formula, bigger sample could help more for generalizability of the results.

## Conclusion

Although CPT is recommended for the treatment of PTSD, it is infrequently delivered,^18^ it seems that CPT can be effective for some symptoms of depression, domestic violence and PTSD. Considering the high prevalence of domestic violence in the world and its high physical and psychological impacts, controlling it by providing comprehensive programs is recommended as the first line of action. 

**Acknowledgments: **We would like to extend our sincere gratitude to the Deputy of Research at Kerman University of Medical Sciences for their invaluable support of this study. (97000823).

**Author Contributions: **M.ShJ. was responsible for data collection and the first draft, M.Gh. handled data analysis, AA.H served as the biostatistics consultant, and A.A. oversaw the final draft and supervised the counseling sessions.
